# Prevention of radiotherapy induced enteropathy by probiotics (PREP): protocol for a double-blind randomized placebo-controlled trial

**DOI:** 10.1186/s12885-021-08757-w

**Published:** 2021-09-16

**Authors:** Yeon Joo Kim, Jesang Yu, Sung Pyo Park, Seung Hae Lee, Young Seok Kim

**Affiliations:** 1 Department of Radiation Oncology, Asan Medical Center, University of Ulsan College of Medicine, 88 Olympic-Ro 43-Gil, Songpa-Gu, Seoul, 05505 Republic of Korea; 2 Department of Ophthalmology, Kangdong Sacred Heart Hospital, Hallym University, College of Medicine, Gangdong-Gu, Seoul, 05355 Republic of Korea

**Keywords:** Radiotherapy, Enteropathy, Probiotics, Efficacy, Safety

## Abstract

**Background:**

Radiation induced enteropathy is a common complication of radiotherapy for pelvic tumors and adversely affects patient quality of life. Probiotics are thought to restore bowel microflora to optimal levels and reinforce intestinal barrier capacity. Although probiotics are effective in the treatment of radiation induced enteropathy, less is known about their efficacy to prevent radiation induced enteropathy.

**Methods:**

This double-blind randomized placebo-controlled study will investigate the efficacy of probiotics to prevent radiation-induced enteropathy in patients with gynecologic or urologic cancer who received pelvic radiotherapy. The study is designed to enroll 248 eligible patients, who will be randomized 1:1 to a probiotic or placebo group. Toxicities will be evaluated using Common Terminology Criteria for Adverse Events (CTCAE) v5.0.

**Discussion:**

The primary aim of this study is to provide high level evidence for the ability of probiotics to prevent acute radiation induced enteropathy. The secondary aims are to determine the effects of probiotics on the incidence of chronic radiation induced enteropathy and the safety of probiotics in patients with gynecologic or urologic cancer.

**Trial registration:**

ClinicalTrials.gov (NCT03978949, Registered on 7 June 2019).

**Supplementary Information:**

The online version contains supplementary material available at 10.1186/s12885-021-08757-w.

## Background

Acute radiation induced enteropathy (RIE), which occurs within 3 months of treatment, has been reported in 60–80% of patients who receive pelvic radiotherapy [1]. Typical symptoms of RIE include diarrhea, nausea, vomiting, and belly cramps. These symptoms may cause dehydration, electrolyte imbalance, and malnutrition, which can adversely affect patient condition at the time of treatment. Treatment interruption or changes in the initial treatment plan may be required, which could compromise the likelihood of tumor control. Delayed RIE can also occur in long-term survivors of pelvic radiotherapy and may affect their quality of life [2].

The World Health Organization (WHO) has defined probiotics as “live micro-organisms which, when consumed in adequate amounts, confer a health benefit on the host” [3]. Probiotics have various effects on their hosts, including the production of antimicrobial bacteriocin and short chain fatty acids, lowering of gut pH, nutrient competition, stimulation of mucosal barrier function, and immunomodulation, although their exact mechanisms of action remain unclear [4]. Probiotics may help prevent and manage RIE by reducing the apoptosis of intestinal epithelial cells, promoting recovery from radiation damage, and enhancing local or systemic immune response against pathogens [5–9]. Two meta-analyses also reported that probiotics may be beneficial for RIE [10, 11]. However, these studies had several limitations. Most were small in size, containing relatively small numbers of patients. Although the largest study included 482 patients from Italy [6], there was no information about chemotherapy, with patients starting probiotics on the first day of radiotherapy, preventing the accumulation of live bacteria prior to treatment.

There are increasing interests in the relation between radiotherapy and the gut microbiome with recent publications of review articles [12, 13]. Both review articles expected that the probiotics could minimize the adverse effect, but pointed out that further robust randomized controlled trials are required. Herein, we plan the present Prevention of Radiotherapy induced Enteropathy by Probiotics (PREP) trial to establish high level evidence.

## Methods/design

### Study aim and design

The PREP study is a prospective, double-blinded, placebo-controlled, randomized trial ongoing at Asan Medical Center, Seoul, Korea. The primary aim of the study is to investigate the efficacy of probiotics, starting 2 weeks before radiotherapy, to prevent acute RIE in patients being treated with pelvic radiotherapy for gynecologic or urologic malignancies. Secondary aims include evaluations of the effects of probiotics on the incidence of chronic RIE and the safety of probiotics in patients with gynecologic or urologic cancer.

**Fig. 1:**
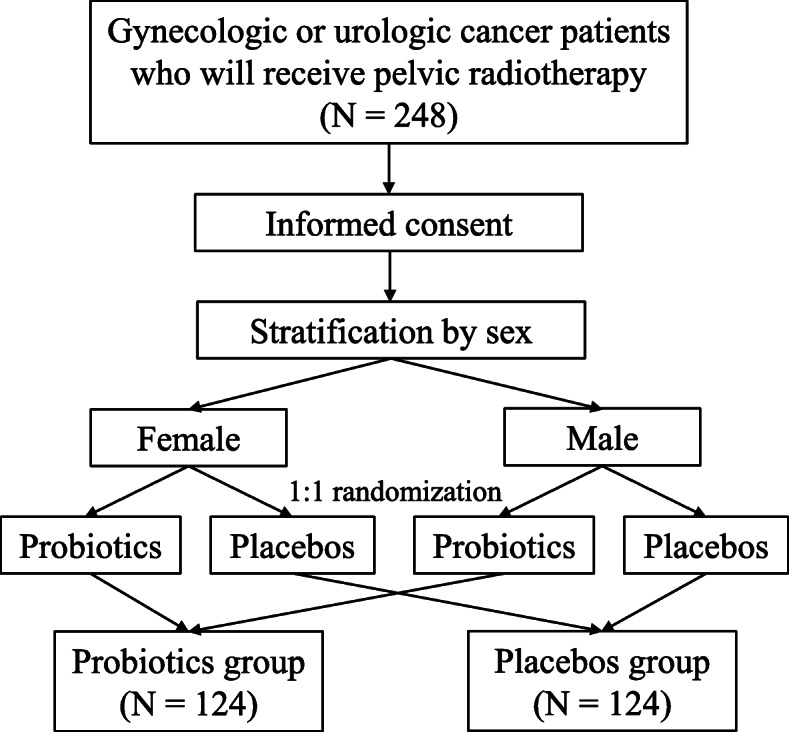
PREP study schema

The design of the PREP trial is illustrated in Fig. 1. Patients diagnosed with gynecologic or urologic malignancies and scheduled to undergo pelvic radiotherapy will be screened and provided with an appropriate explanation about the PREP trial. Written informed consent will be obtained by radiation oncologists from patients who elect to participate in the study. After stratification by sex, participants will be randomly assigned 1:1 to the probiotics or placebo group using blocked randomization. Sex will be the single stratification factor, as patients with gynecologic malignancies have a greater tendency to require concurrent chemoradiotherapy, which affects the incidence of RIE. The block size and sequence for randomization will vary. The randomization sequence will be generated by a central web-based computer so that allocation will be concealed from the primary investigator who enrolls the patients. Neither participants nor physicians will be aware of their group assignments.

### Inclusion criteria

Patients will be included if they [1] have histologically proven malignancies of the uterus, ovary, vagina, vulva, bladder, urethra, or prostate [2]; are aged > 20 years [3]; have an Eastern Cooperative Oncology Group performance score of 0–2 [4]; have an adequate hematologic condition within 6 months after enrollment, defined as absolute neutrophil count ≥1500 cells/mm^3^, platelet count ≥50,000 cells/mm^3^, and hemoglobin ≥8.0 g/dl [5]; have appropriate kidney function within 6 months after enrollment, defined as creatinine < 2.0 ng/dL; and [6] have appropriate liver function within 6 month after enrollment, defined as total bilirubin < 1.5 times the maximum normal value and alanine aminotransferase and aspartate aminotransferase < 2.5 times the maximum normal value.

### Exclusion criteria

Patients will be excluded if they have a previous history of pelvic radiotherapy, double primary cancer other than skin or thyroid cancer, or severe comorbidity, or if they had participated in another clinical trial within 1 month.

### Dropout criteria

Patients who withdraw consent will be dropped out of the study, as will patients with any medical issues or unexpected toxicities, defined as grade ≥ 3 toxicities based on Common Terminology Criteria for Adverse Events (CTCAE) v5.0 occurring during treatment.

### Treatment implementation

Patients allocated to the probiotics group will be administered two Biscanen capsules (*Bacillus licheniformis* 250 mg containing 250 million colony forming units) three times per day, starting 2 weeks before the initial day of pelvic radiotherapy to the final day of radiotherapy. Patients in the placebo group will be administered placebo pills according to the same schedule.

Prior to radiotherapy, all patients will undergo enhanced computed tomography (CT) scans at 2.5 mm thickness. Participants should be relaxed and in a supine position facilitated by immobilization devices. Simulation CT images will be fused with the results of diagnostic magnetic resonance imaging or positron emission tomography-computed tomography in patients who require delineation of the prostate or gross target volume. Clinical target volume (CTV) will include pelvic regional nodal areas, based on the Asan Medical Center protocol for each tumor type. The upper margin for pelvic radiotherapy will be the junction of the 4th and 5th lumbar spine. The planning target volume (PTV) will include the CTV with 3–10 mm margins. Organs at risk, including the small bowel, large bowel, bladder, rectum, and femoral head, will be delineated according to the guidelines of the Radiation Therapy Oncology Group.

All patients will receive intensity modulated radiotherapy (IMRT) with the PTV required to be irradiated with ≥95% of the prescribed dose. Dose constraints will include the following: 1) 30% of the entire volume of the bowels (small and large) must not receive more than 40 Gy irradiation; 2) 60% of the volume of the rectum must receive ≤40 Gy; 3) 35% of the bladder volume must receive ≤45 Gy (except in patients with bladder cancer); and 4) 15% of the femoral head volume must receive < 35 Gy. Quality assurance will be performed before radiotherapy in all patients. During each treatment, cone-beam CT will be performed for image guidance. Radiotherapy will be scheduled to begin within 10 days after CT simulation, with the total radiotherapy period not exceeding 10 weeks.

### Data collection and follow-up period

**Fig. 2:**
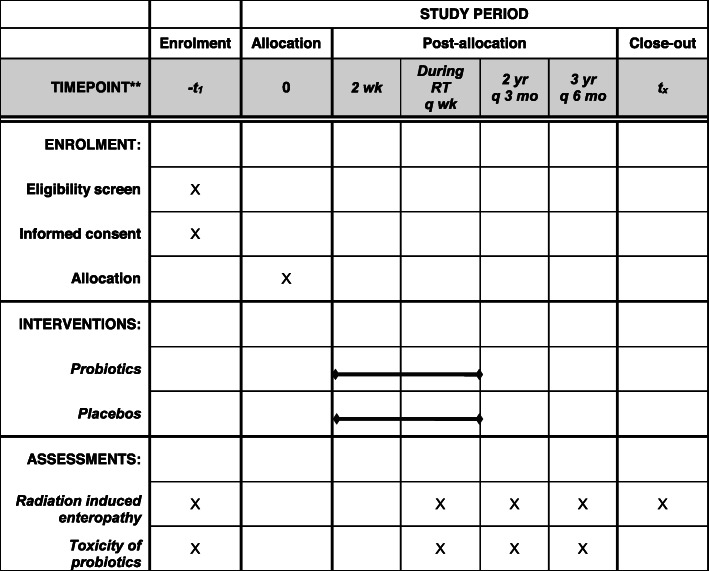
Intervention and assessment schedule for the PREP trial according to the recommendations for interventional trials (SPIRIT)

The primary endpoint will be the incidence of acute RIE, and the secondary endpoints will be chronic RIE and toxicities due to probiotics. Patient enrollment and treatment is expected to be completed within 2 years. Patient data on endpoints will be collected before the start of radiotherapy, every week during radiotherapy, every 3 months for the first 2 years after radiotherapy, and every 6 months subsequently for up to 5 years after radiotherapy (Fig. 2). All toxicities will be graded using CTCAE v5.0. The trial design and protocol adhere to the Recommendations for Interventional Trials (SPIRIT) criteria. The SPIRIT checklist and figure can be found in Supplemental Table S1.

There will be interim analysis for toxicities but not for efficacy at the timing when 50% of target subjects will be enrolled. The trial will be terminated early if the odds ratio of acute toxicities grade over 2 is more than 2 between the two groups. To protect patient data, each patient will be assigned a separate identification code. All data will be stored in password-accessible files, available only to investigators. All data will be deleted 3 years after the end of the study.

### Sample size and statistical analysis

Two proportion test in Power Analysis and Sample Size Software 2018 (NCSS, LLC. Kaysville, Utah, USA) was utilized to estimate the sample size. Based on the assumption that 30% of the patients in the placebo group and 15% of the patients in the probiotics group will experience grade ≥ 2 acute toxicities, 118 patients per group will be required to show a significant effect based on an alpha value of 0.05 and a beta of 0.2. Assuming a 5% dropout rate, 124 patients per group will be required (total, 248 patients).

Acute toxicities will be analyzed in the intention to treat population, with categorical variables in the two groups compared by the Chi-Square or Fisher’s exact test. To find the other confounding factors for RIE, univariate and multivariate logistic regression for acute toxicity will be performed. The times from the start of radiotherapy to acute grade ≥ 2 toxicities and to any chronic toxicities will be calculated using the Kaplan-Meier method and compared in the two groups using the log-rank test to compare survival curves between two groups. In the whole population, multivariate analysis, Cox proportional hazard model will be utilized to find the significant factor for the incidence of RIE in addition to the use of probiotics. Factors with *p* ≤ 0.2 in log-rank test will be entered in multivariate analysis. Descriptive statistics of all analyzed parameters will be provided, whenever appropriate.

## Discussion

Radiotherapy is utilized in about 50% of cancer patients [1], and its role is especially crucial in patients with gynecologic and genitourinary malignancies. Radiation is required to manage 80–90% of women with carcinoma cervix, 60% of women with endometrial cancer, and 50% of women with carcinoma vulva [14]. Radiotherapy is a major treatment option for every stage of prostate cancer [15] and has been adapted for bladder cancer patients who want bladder preservation [16]. However, 60–80% of patients who receive pelvic radiotherapy experience acute RIE [1], which may lead to late RIE and affect the quality of life of long-term cancer survivors. The Post Operative Radiation Therapy in Endometrial Cancer (PORTEC-2) trial demonstrated that diarrhea was significantly more frequent in patients who did than did not receive pelvic radiotherapy, indicating that treated patients must remain close to a toilet, reducing their social functioning and quality of life [17]. Although high precision radiotherapy techniques such as IMRT have been widely introduced to reduce irradiation of the bowels, about 50% of patients who received pelvic IMRT experience acute gastrointestinal toxicities of any grade [18], and 34% of patients suffer from frequent or almost constant severe diarrhea [19].

Several studies have evaluated the usefulness of probiotics in patients with infectious diarrhea and antibiotic-induced diarrhea [20]. Probiotics are also used to control inflammatory bowel disease (IBD) as gut dysbiosis is regarded as having a potential pathogenic role in IBD [21]. Although the precise mechanisms by which probiotics affect pathogenic situations remain unclear, several hypotheses have been suggested, including restoration of microbial balance, modulation of mucosal protection, protection against pathogens including protective immune responses through immunization, and modification of gut-associated lymphoid cells [22]. Irradiation of the bowels induces cell death in the rapidly proliferating crypt epithelium and protracted acute inflammatory reactions in the lamina propria. Crypt cell death leads to lack of replacement of the villus epithelium, breakdown of the mucosal barrier, and mucosal inflammation [1]. Probiotics have also shown potential in relieving gastrointestinal inflammation in IBS patients during and after radiotherapy [10].

Most studies evaluating the effects of probiotics for RIE have reported that probiotics reduce acute diarrhea [5, 6, 23–25] (Table 1). Most of these studies, however, included small numbers of patients, and only one study started patients on probiotics 1 week before the start of radiotherapy. The beneficial effects of probiotics are thought to be proportional to the concentration of live bacteria [25], but less is known about the optimal period to take probiotics that results in a sufficient concentration of live bacteria. However, probiotics may be less effective when started at the initiation of radiotherapy than beforehand. Thus, the present PREP study is designed so that probiotics will be administered 2 weeks prior to the initiation of radiotherapy.

**Table 1:** Characteristics of previous randomized controlled trials studying the effects of probiotics on radiation induced enteropathy

First author	Year /Country	No. of patients Pl/Pr	Primary tumor	Total RT dose (Gy)	Radiation technique	Chemotherapy Pl/Pr (%)	Probiotics	Daily CFU	Timing	Toxicity scoring system	Acute diarrhea (%) Pl/Pr
Linn	2018/ Myanmar	28/26	Cervical	50	2D	79/73	Lactobacillus, Bifidobacterium	6 × 10^9^	During RT, t.i.d.	CTCAE 4.0	Mild–moderate 82/54 (SS)
Demers	2014/ Canada	86/140	Gynecologic, rectal, prostate	40–50.4	NA	56/54 or 45	Lactobacillus, Bifidobacterium	2.6 × 10^9^ or 3 × 10 ^10^	During RT, b.i.d. or t.i.d.	WHO or CTCAE 3.0	Moderate–severe 83/65 (SS)
Chitapanarux	2010/ Thailand	31/32	Cervical	40–56	2D	100	Lactobacillus, Bifidobacterium	4 × 10^9^	1 week before RT, during RT, b.i.d.	CTCAE 2.0	Grade 2–3 45/9 (SS)
Giralt	2008/ Spain	41/44	Cervical, endometrial	40–50	NA	For cervical cancer	Streptococcus, Lactobacillus	3 × 10^8^	1 week, t.i.d.	CTCAE	Grade ≥ 2 59/68 (NS)
Delia	2007/ Italy	239/243	Sigmoid, rectal, cervical	60–70	NA	NA	Lactobacillus, Bifidobacterium, Streptococcus	1.35 × 10^12^	During RT, t.i.d.	WHO	Any grade 52/32 (SS)

*2D* two-dimensional; *CFU* colony forming units; *CTCAE* Common Terminology Criteria for Adverse Events; *No*. number; *NA* not available; *NS* not significant; *Pl* placebo; *Pr* Probiotics; *RT* radiotherapy; *SS* statistically significant; *WHO* World Health Organization

The largest prospective double-blind, placebo-controlled trial performed to date on the relationship between probiotics and RIE [6] found that the incidence of radiation induced diarrhea was significantly lower in the probiotics than in the placebo group (31.6% vs. 51.8%, *P* < 0.001), as was the incidence of grade 3 or 4 diarrhea (1.4% vs. 55.4%, *P* < 0.001). That study, however, did not provide information on chemotherapy, one of the major factors contributing to RIE, nor did it evaluate the incidence of chronic RIE. Furthermore, IMRT is frequently utilized in real-world clinical conditions for pelvic radiotherapy, suggesting the need to evaluate the effects of probiotics in patients undergoing IMRT.

Probiotics are generally considered safe in a healthy population [20]. However, it is essential to investigate the safety of probiotics in immunocompromised cancer patients. Case studies have reported that the probiotics Lactobacillus and Bacillus may cause infection in these patients, resulting in sepsis [26, 27]. However, a systematic review of the use of probiotics in cancer patients demonstrated that sepsis due to probiotics was rare [28]. The inclusion criteria of the PREP trial will filter out severely immunocompromised patients. Moreover, the strain of *Bacillus licheniformis* that will be used in the PREP trial has never shown toxicity in humans, but has reduced the incidence of diarrhea associated with antibiotic therapy [29]. One in vivo study suggested that this probiotic may carry a risk of antibiotic resistance [30].

The purpose of the PREP study is to provide high-level evidence on the use of probiotics for reducing the incidence of RIE. We anticipate that the rate of acute RIE will be significantly lower in the probiotic than in the placebo arm. We also expect that probiotics will decrease the incidence of chronic RIE and will have little or no toxicity in these patients.

## Trial status

Patient recruitment was started on 1 June 2019 and is currently ongoing based on protocol version 1.6.

Recruitment is expected to be complete by 1 June 2021.

## Supplementary Information

**Additional file 1.**

## Data Availability

The datasets analyzed during the current study will be available from the corresponding author on reasonable request.

## Abbreviations

CT: Computed tomography
CTCAE: Common terminology criteria for adverse events
CTV: Clinical target volume
IBD: Inflammatory bowel disease
IMRT: Intensity modulated radiotherapy
PTV: Planning target volume
RIE: Radiation induced enteropathy
WHO: World health organization

## References

[1] Hauer-Jensen M, Denham JW, Andreyev HJN (2014). Radiation enteropathy--pathogenesis, treatment and prevention. Nat Rev Gastroenterol Hepatol.

[2] Abayomi J, Kirwan J, Hackett A (2009). The prevalence of chronic radiation enteritis following radiotherapy for cervical or endometrial cancer and its impact on quality of life. Eur J Oncol Nurs.

[3] Hotel ACP, Cordoba A (2001). Health and nutritional properties of probiotics in food including powder milk with live lactic acid bacteria. Prevention..

[4] Kechagia M, Basoulis D, Konstantopoulou S, Dimitriadi D, Gyftopoulou K, Skarmoutsou N (2013). Health benefits of probiotics: a review. ISRN Nutr.

[5] Demers M, Dagnault A, Desjardins J (2014). A randomized double-blind controlled trial: impact of probiotics on diarrhea in patients treated with pelvic radiation. Clin Nutr.

[6] Delia P, Sansotta G, Donato V, Frosina P, Messina G, De Renzis C (2007). Use of probiotics for prevention of radiation-induced diarrhea. World J Gastroenterol.

[7] Urbancsek H, Kazar T, Mezes I, Neumann K (2001). Results of a double-blind, randomized study to evaluate the efficacy and safety of Antibiophilus in patients with radiation-induced diarrhoea. Eur J Gastroenterol Hepatol.

[8] Fuccio L, Guido A, Eusebi LH, Laterza L, Grilli D, Cennamo V, Ceroni L, Barbieri E, Bazzoli F (2009). Effects of probiotics for the prevention and treatment of radiation-induced diarrhea. J Clin Gastroenterol.

[9] Guandalini S (2011). Probiotics for prevention and treatment of diarrhea. J Clin Gastroenterol.

[10] Liu MM, Li ST, Shu Y, Zhan HQ (2017). Probiotics for prevention of radiation-induced diarrhea: a meta-analysis of randomized controlled trials. PLoS One.

[11] Gibson RJ, Keefe DM, Lalla RV, Bateman E, Blijlevens N, Fijlstra M (2013). Systematic review of agents for the management of gastrointestinal mucositis in cancer patients. Support Care Cancer.

[12] Oh B, Eade T, Lamoury G, Carroll S, Morgia M, Kneebone A, et al. The gut microbiome and gastrointestinal toxicities in pelvic radiation therapy: a clinical review. Cancers. 2021;13(10):2353. 10.3390/cancers13102353. 10.3390/cancers13102353 PMC8153110 34068216

[13] Liu J, Liu C. Yue JJRO, Radiotherapy and the gut microbiome: facts and fiction. Cancers. 2021;16(1):1–15. 10.1186/s13014-020-01735-9. 10.1186/s13014-020-01735-9 PMC7805150 33436010

[14] Dasari P, Vivekanandam S, Raghava KSA. Radiation for Gynaecological malignancies. Radiotherapy. 2017;63. 10.5772/67202.

[15] Network NCC. NCCN clinical practice guidelines in oncology, prostate cancer—version 4.2019. 2019.

[16] Kim YJ, Byun SJ, Ahn H, Kim C-S, Hong B-S, Yoo S, Lee JL, Kim YS (2017). Comparison of outcomes between trimodal therapy and radical cystectomy in muscle-invasive bladder cancer: a propensity score matching analysis. Oncotarget..

[17] Nout RA, Putter H, Jürgenliemk-Schulz IM, Jobsen JJ, Lutgens LCHW, van der Steen-Banasik EM, Mens JWM, Slot A, Stenfert Kroese MC, Nijman HW, van de Poll-Franse LV, Creutzberg CL (2012). Five-year quality of life of endometrial cancer patients treated in the randomised post operative radiation therapy in endometrial Cancer (PORTEC-2) trial and comparison with norm data. Eur J Cancer.

[18] Joo JH, Kim YJ, Kim YS, Choi EK, Kim JH, Lee S-W, Song SY, Yoon SM, Kim SS, Park JH, Jeong Y, Ahn H, Kim CS, Lee JL, Ahn SD (2013). Whole pelvic intensity-modulated radiotherapy for high-risk prostate cancer: a preliminary report. Radiat Oncol J.

[19] Klopp AH, Yeung AR, Deshmukh S, Gil KM, Wenzel L, Westin SN, Gifford K, Gaffney DK, Small W, Thompson S, Doncals DE, Cantuaria GHC, Yaremko BP, Chang A, Kundapur V, Mohan DS, Haas ML, Kim YB, Ferguson CL, Pugh SL, Kachnic LA, Bruner DW (2018). Patient-reported toxicity during pelvic intensity-modulated radiation therapy: NRG oncology-RTOG 1203. J Clin Oncol.

[20] Nomoto K (2005). Prevention of infections by probiotics. J Biosci Bioeng.

[21] Ganji-Arjenaki M, Rafieian-Kopaei M (2018). Probiotics are a good choice in remission of inflammatory bowel diseases: a meta analysis and systematic review. J Cell Physiol.

[22] Fedorak RN, Madsen KL (2004). Probiotics and the management of inflammatory bowel disease. Inflamm Bowel Dis.

[23] Linn YH, Thu KK, Win NHH (2019). Effect of probiotics for the prevention of acute radiation-induced diarrhoea among cervical cancer patients: a randomized double-blind placebo-controlled study. Probiotics Antimicrobial Proteins.

[24] Chitapanarux I, Chitapanarux T, Traisathit P, Kudumpee S, Tharavichitkul E, Lorvidhaya V (2010). Randomized controlled trial of live lactobacillus acidophilus plus bifidobacterium bifidum in prophylaxis of diarrhea during radiotherapy in cervical cancer patients. Radiat Oncol.

[25] Giralt J, Regadera JP, Verges R, Romero J, de la Fuente I, Biete A, Villoria J, Cobo JM, Guarner F (2008). Effects of probiotic Lactobacillus casei DN-114 001 in prevention of radiation-induced diarrhea: results from multicenter, randomized, placebo-controlled nutritional trial. Int J Radiat Oncol Biol Phys.

[26] Land MH, Rouster-Stevens K, Woods CR, Cannon ML, Cnota J, Shetty AK (2005). Lactobacillus sepsis associated with probiotic therapy. Pediatrics..

[27] Richard V, Van Der Auwera P, Snoeck R, Daneau D, Meunier F (1988). Nosocomial bacteremia caused byBacillus species. Eur J Clin Microbiol Infect Dis.

[28] Redman M, Ward E, Phillips R (2014). The efficacy and safety of probiotics in people with cancer: a systematic review. Ann Oncol.

[29] Horosheva TV, Vodyanoy V, Sorokulova I. Efficacy of Bacillus probiotics in prevention of antibiotic-associated diarrhoea: a randomized, double-blind, placebo-controlled clinical trial. JMM Case Rep. 2014;1(3). 10.1099/jmmcr.0.004036.

[30] Sorokulova IB, Pinchuk IV, Denayrolles M, Osipova IG, Huang JM, Cutting SM, Urdaci MC (2008). The safety of two Bacillus probiotic strains for human use. Dig Dis Sci.

